# Photovoltaic Glass Waste Recycling in the Development of Glass Substrates for Photovoltaic Applications

**DOI:** 10.3390/ma16072848

**Published:** 2023-04-03

**Authors:** Karina Treviño Rodríguez, Astrid Iriana Sánchez Vázquez, Juan Jacobo Ruiz Valdés, Jorge Ibarra Rodríguez, María Guadalupe Paredes Figueroa, Samuel Porcar García, Juan Bautista Carda Castelló, Anabel Álvarez Méndez

**Affiliations:** 1 Laboratorio de Materiales III, División de Estudios de Posgrado, Facultad de Ciencias Químicas, Universidad Autónoma de Nuevo León, Guerrero y Progreso S/N, Col. Treviño, C.P., Monterrey 64570, Mexico; karina.trevinord@uanl.edu.mx (K.T.R.); astrid.sanchezvz@uanl.edu.mx (A.I.S.V.);; 2 Universidad de Monterrey, Av. Ignacio Morones Prieto 4500-Pte, Zona Valle Poniente, San Pedro Garza García 66238, Mexico; 3 Department of Inorganic and Organic Chemistry, Universitat Jaume I, 12071 Castellon de la Plana, Spain

**Keywords:** photovoltaic waste glass, substrate, recycling, photovoltaics

## Abstract

Because of the increasing demand for photovoltaic energy and the generation of end-of-life photovoltaic waste forecast, the feasibility to produce glass substrates for photovoltaic application by recycling photovoltaic glass waste (PVWG) material was analyzed. PVWG was recovered from photovoltaic house roof panels for developing windows glass substrates; PVWG was used as the main material mixed with other industrial waste materials (wSG). The glass was casted by air quenching, annealed, and polished to obtain transparent substrates samples. Fluorine-doped tin oxide (FTO) was deposited as back contact on the glass substrates by spray pyrolysis. The chemical composition of the glass materials was evaluated by X-ray fluorescence (XRF), the thermal stability was measured by differential thermal analysis (DTA) and the transmittance was determined by UV-VIS spectroscopy. The surface of the glass substrates and the deposited FTO were observed by scanning electron microscopy (SEM), the amorphous or crystalline state of the specimens were determined by X-ray diffraction (XRD) and the sheet resistance was evaluated by the four-point probe method. The sheet resistance of the deposited FTO on the wSG substrate was 7.84 ± 3.11 Ω/□, lower than that deposited on commercial soda-lime glass (8.48 ± 3.67 Ω/□), meaning that this material could present improved conduction of the produced electrons by the photovoltaic effect. This process may represent an alternative to produce glass substrates from waste materials that could be destined for photovoltaic applications, especially the production of ecological photovoltaic windows.

## 1. Introduction

Photovoltaic energy is one of the most promising sources of renewable energy, since it allows the generation of electricity without generating greenhouse gases during its operation [1]. According to the International Renewable Energy Agency (IRENA) [2] the photovoltaic energy capacity installed worldwide is around 480 GW, and it is forecasted an increase to 8519 GW by 2050. Photovoltaic panels have an average lifespan of 25 to 30 years [3] therefore, the growing generation of solar panel waste is predicted. The accumulation of 8 million tons of waste from photovoltaic systems is forecasted worldwide by 2030 and 78 million tons for the year 2050 [3]. According to Peplow’s forecast [4] 80 million of metric tons around the world will be generated by 2050. For the case scenario of Mexico, according to Domínguez et al. [1], it is predicted that 690,907 tons of photovoltaic waste will be generated by 2045.

Photovoltaic wastes are multi-material composites that contain diverse materials, such as, glass, metal rods and plastic; the amount of these materials on the photovoltaic waste depends on the type of solar panel [5]. However, crystalline silicon cells panels are the dominant waste in the generation of photovoltaic residues [6]. This kind of solar panel waste contains materials with high commercial value such as aluminum, copper, silicon, and silver, however, the glass represents around 75% [4]—80% [3] of the total mass of the photovoltaic waste.

Diverse pathways of solar panel waste glass recycling have been proposed; the most common is its reincorporation to the solar panel production [7,8]. Other proposed methods of recycling consist of mixing this waste with other residues in the production of different products, such as glass fiber [3,9] clay bricks [10,11] glass-ceramic materials [12,13] and zeolites [14].

Although these processes represent alternatives for the recycling of this waste, one of the current technological challenges is the development of products with additional functions that contribute to reduce the greenhouse gas emissions and mitigate climate change effects. The photovoltaic technology is key to enabling the successful struggle against this worldwide challenge [15,16], hence, an example of these materials are the Building Integrated Photovoltaics (BIPV), which are materials such as windows, facades and tiles with dual functionality: they can be used to replace the conventional elements of construction and generate energy [17,18]. BIPV materials are currently on the market worldwide; some examples are the photovoltaic glass from the company Onix Solar^®^ (Vicolozano, Spain) [19] and the photovoltaic roof tiles from Tesla^®^ (Buffalo, NY, USA) [20]. BIPV materials have high potential because they reduce the required pace for their installation [21] compared to that of conventional solar panels.

One of the BIPVs developed and studied are the photovoltaic windows, which are made by the integration of photovoltaic technology in traditional windows. Their main advantage is the production of electricity by means of the photovoltaic effect, also, compared with traditional windows, photovoltaic windows can attenuate the solar radiation penetrating rooms [22,23], thereby reducing the power consumption of air-conditioning systems [24]. Soda-lime glass (SLG) is one of the most used substrates materials for the development of photovoltaic windows due to its transparency, high volume, and low-cost production [25]. Due to the increasing demand of photovoltaic technology, it is important to incorporate waste material to the development of photovoltaic products. There is some research about the incorporation of waste material in the development of ceramic substrates, such as the works of Becerril-Romero et al. [26] and Fraga et al. [27,28], in which photovoltaic devices were deposited on ceramic tiles synthesized from clay with glass waste, metallurgical slag and ceramic waste; however, to our knowledge, no vitreous substrates from waste materials have been reported for the development of photovoltaic windows. This is considered mandatory because the solar industry’s demand for glass is growing [7,29,30].

The purpose of this work was the production of glass substrates using PVWG as main material, as well as other residues such as dolomite and quartz sand, and applying a transparent conductive oxide (TCO) in order to evaluate their properties for their possible application in thin-film photovoltaic glass windows.

## 2. Materials and Methods

The overall methodology of this study is summarized in three processes: the first process consisted of the recovery of clean PVWG from the photovoltaic waste. The second process encompassed the preparation of glass substrates by glass casting, cutting, and polishing steps, and the third process consisted of the FTO deposition on the produced substrates.

Polycrystalline silicon photovoltaic panel waste was received and treated to recover clean photovoltaic waste glass (PVWG), and it was separated from metal rods, Tedlar^®^, silicon cells and ethyl-vinyl acetate (EVA). For this process, the Kang et al. procedure [31] was followed, however, in this work, instead of using organic solvents, a heat treatment at 220 °C was carried out in order to manually separate the Tedlar^®^ sheets from PVWG, Si cells and metallic contacts. Once the Tedlar^®^ was separated, the PVWG was heated to 550 °C in order to evaporate EVA, and the PVWG was manually separated from the Si-cells.

The first batch was prepared in order to produce a glass substrate only with PVWG, on the other hand, the batch for ecological glass substrates (wSG) was formulated to reach three objectives: to preserve the PVWG as the main material, to incorporate another waste materials, and to increase the SiO_2_, CaO and MgO content in comparison to that of a typical SLG formulation. This is mainly due to reports of the various effects of sodium diffusion from glass substrates in the deposition processes of conductive and absorber layers [18]. Several researchers reported that the glass of solar panelshave the same composition of that of the SLG with low Fe content [32]. To obtain a glass substrate, PVWG was used with other industrial waste materials previously studied by Alvarez-Mendez et al. [33], dolomite out of specifications from Industrias Peñoles and rejected quartz sand from Grupo Materias Primas de México were incorporated. Table 1 indicates the proportions of the waste materials.

The glass was obtained throughout the process of heating the batch mixture in alumina crucibles at 1450 °C for 2 h; the product was air quenched, to refine the glass and eliminate air bubbles and the glass was re-heated at 1450 °C in platinum crucibles and air quenched in rectangular steel molds. The glass pieces were annealed at 517 °C by 1 h, thereafter they were cut and polished to obtain flat transparent 3 mm-thick glass pieces, similar to domestic SLG windows.

Fluoride-doped tin oxide (FTO) was selected as the front contact layer and deposited onto the glass substrates using the spray pyrolysis technique [34], FTO is a transparent conductive oxide, that consists in fluoride doped tin oxide (SnO_2_:F), which is widely used due to its capacity to conduct the produced electrons in the photovoltaic effect. The precursor solution was prepared according to the procedure presented by Koirala et al. [35]. Tin tetrachloride pentahydrate (99.99% Sigma-Aldrich, Wuxi, China) was used as Sn source and diluted in ethanol (99%, Scharlab, Barcelona, Spain), on the other hand, NH_4_F (98% Alfa Aesar, Kandel, Germany) was used as F source and diluted in distilled water, the precursor solution was then ultrasonicated for 30 min and placed in the atomizer.

The substrates were heated on a heating plate up to 500 °C, once the temperature was reached, the solution was nebulized and sprayed on the substrates, once the deposition process was completed, the substrates were cooled until they reached room temperature.

The chemical analysis of glass materials was analyzed by X-ray fluorescence (XRF) in a S4 Pioneer–Bruker. X-ray Diffraction was performed in a PANalytical Expert-Pro diffractometer at 45 kV and 40 mA, using radiation CuKα at 1.5406 Å; the samples were analyzed in 2θ from 5° to 90° with a step size of 0.01°. The thermal stability of the glass materials was given by differential thermal analysis (DTA) in a Shimadzu DTA-50 apparatus under dry air atmosphere at 20 mL/min and a heating rate of 10 °C/min from 25 to 1200 °C, using Al_2_O_3_ as reference material. UV-VIS transmittance was measured in an Evolution 300 UV-VIS spectrophotometer, from 300 to 1000 nm, using empty cells as a reference. Electrical sheet resistances of deposited FTO were measured in a OSSILA Four-Point Probe with a target current at 10 mA, and a maximum voltage of 10 V with increments of 0.01 V. The morphology of the synthesized glass substrate surfaces and the sputtered FTO was observed by SEM-EDS with a JEOL JSM-6701f equipment at 15 kV, and a cross section was used to measure the thickness of the deposited FTO with this technique.

## 3. Results

### 3.1. PVWG Separation and Characterization

**Figure 1:**
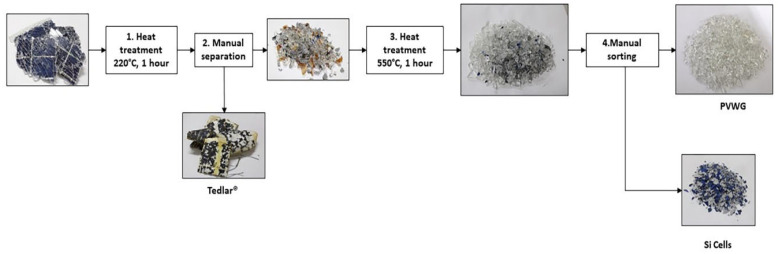
PVWG Recovery pathway.

Figure 1 shows the different materials obtained from photovoltaic waste separation, and Table 2 presents the mass percentage of recovered material. PVWG was recovered in a proportion of 80.83% from the total mass of photovoltaic waste, where 2.93% corresponded to crushed silicon cells with powder PVWG, and 11.95% corresponded to a waste formed by Tedlar^®^, silicon cells, EVA and metal rods, and 4.27% was associated to EVA evaporation.

Table 3 shows the chemical analysis of PVWG and a commercial soda-lime glass (SLG) by XRF. The main components of both glasses are SiO_2_, Na_2_O and CaO.

**Figure 2:**
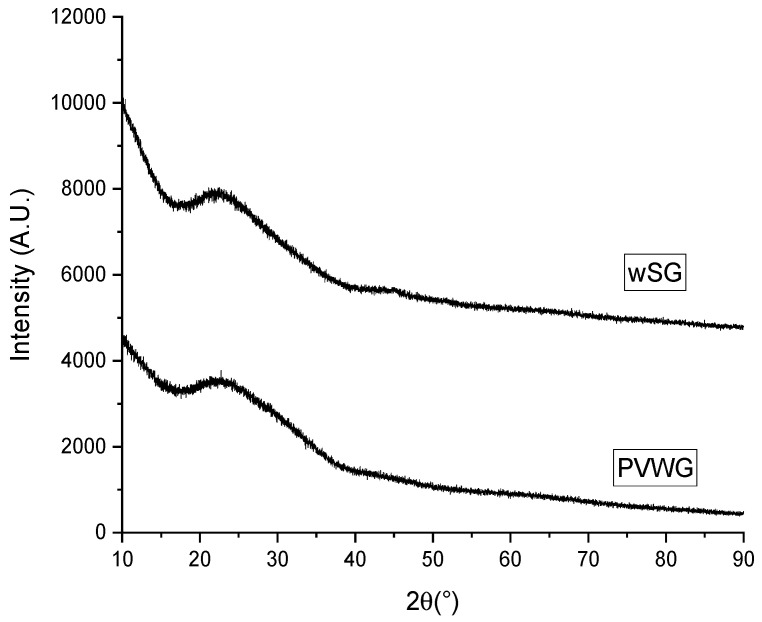
XRD Patterns of PVWG and wSG.

**Figure 3:**
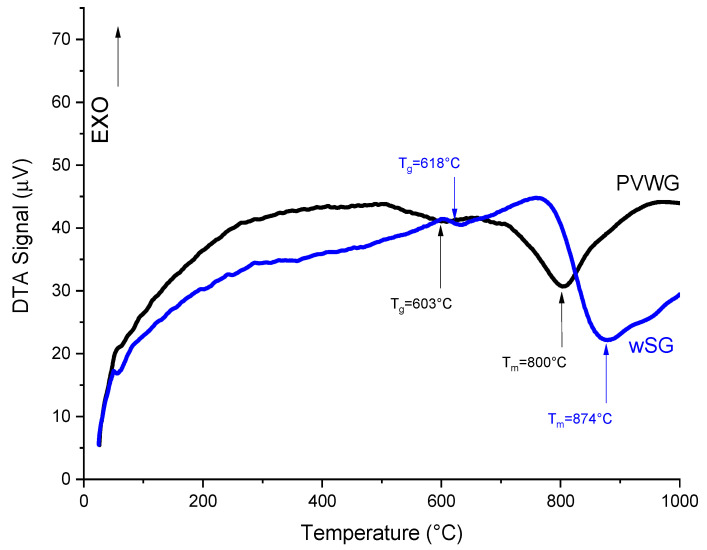
DTA analyses of PVWG and wSG.

Figure 2 displays the XRD pattern of the recovered PVWG, proving that devitrification was not performed in the recovered PVWG after heat treatment, as per the obtained characteristic amorphous pattern of vitreous materials. Furthermore, Figure 3 depicts DTA analysis of this glass exhibiting two thermal events associated to glass transition (T_g_) at 603 °C and a melting point (T_m_) at 800 °C.

### 3.2. Glass Quenching, Substrate Preparation and Characterization

**Figure 4:**
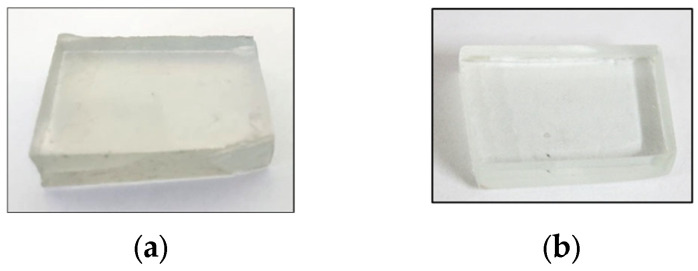
Three (3) mm-thick flat substrates: (**a**) PVWG; (**b**) wSG.

After the quenching, annealing, cutting and polishing processes, transparent rectangular 3 mm-thick flat glass pieces were obtained (Figure 4).

Table 3 presents the chemical composition for wSG analyzed by XRF. The SiO_2_ in this composition was around 71% similar to that of the PVWG; the CaO content increased to 10.68% and Na_2_O decreased to 7.18%, hence this glass formula prepared with another waste material presented a lower proportion of the Na^+^ alkali ion than that found in typical SLG and PVWG.

The XRD pattern of wSG (Figure 2) shows the amorphous glass pattern, discarding devitrification phenomena during the quenching and annealing processes.

The DTA analysis of wSG is shown in Figure 3. A thermal event was found at 618 °C that is associated to T_g_ of this glass, which is higher than that of the PVWG, and the melting point was located at 874 °C.

**Figure 5:**
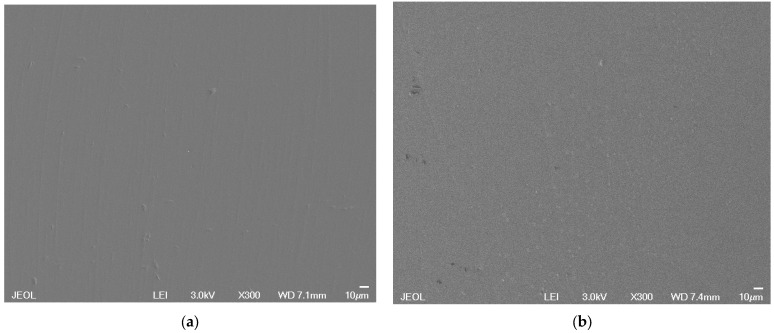
SEM images of the substrates (surface): (**a**) wSG; (**b**) PVWG.

SEM images of PVWG and wSG are shown in Figure 5. A regular surface without the presence of crystals was observed in both substrates. The defects observed could be attributed to the polishing process.

**Figure 6:**
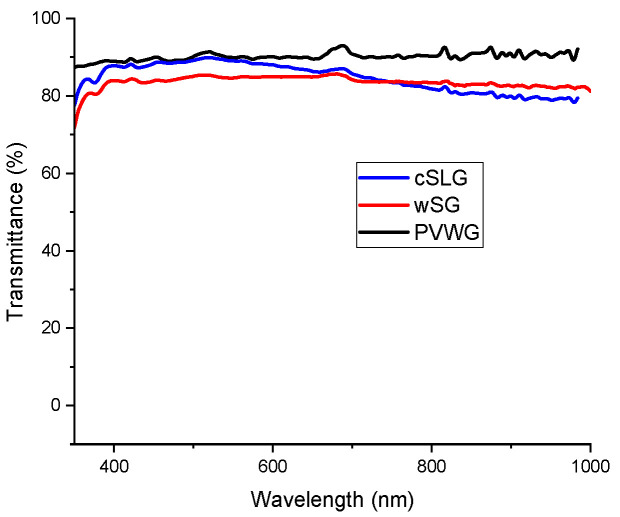
UV-VIS transmittance spectra of PVWG, wSG and cSLG substrates.

Figure 6 depicts the UV-VIS transmittance spectra of PVWG and wSG from 350 to 1000 nm, which were measured and compared with that of the commercial SLG (cSLG). The average transmittance in the region from 350 to 1000 nm for PVWG was 90.15 ± 0.98%, while in wSG was 83.60 ± 1.52%. In the case of cSLG, the transmittance reached around 90% from 425 to 515 nm, and then decreased to 78% at 1000 nm, the mean transmittance for this glass was 84.76 ± 3.60% for the entire analyzed region.

### 3.3. FTO Deposition and Characterization

The sheet resistances for the deposited FTO were 10.13 ± 3.47 Ω/□ for PVWG, 7.84 ± 3.11 Ω/□ for wSG, and 8.48 ± 3.67 Ω/□ for commercial SLG. On these results sheet resistance in wSG was lower than those of the commercial soda lime glass and PVWG substrates, hence this material was more suitable to conduct the produced electrons by the photovoltaic effect.

**Figure 7:**
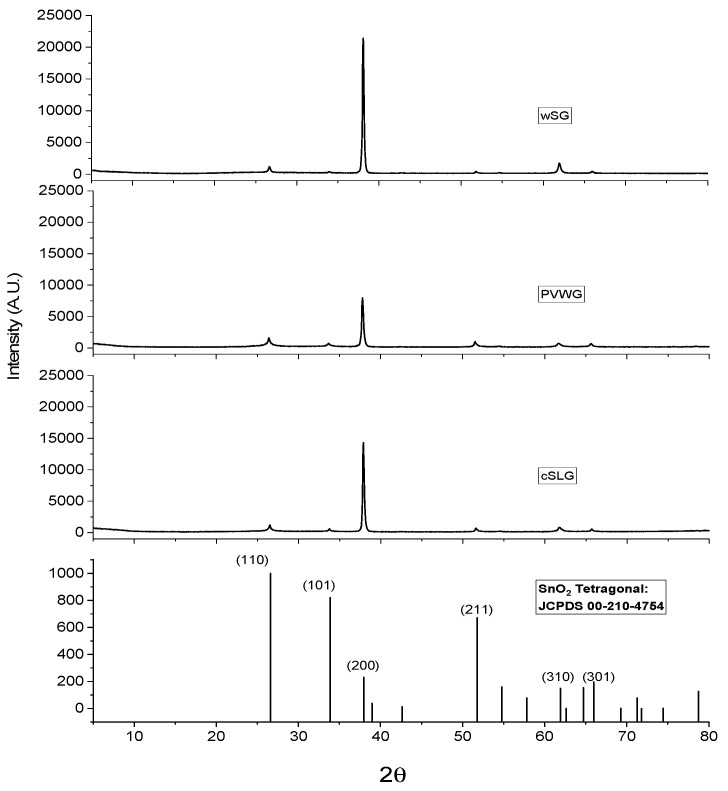
XRD Patterns of deposited FTO on cSLG, PVWG and wSG substrates.

Figure 7 illustrates the XRD pattern of the deposited FTO on commercial SLG, PVWG and wSG substrates. The only detected crystalline phase was the SnO_2_ tetragonal structure (a = 4.7358 Å, c = 3.1873 Å) [JCPDS: 00-210-4754] with the main orientation in the plane (200), planes (110) and (310) are observed with less intensity, while other planes such as (101), (211) and (301) are barely shown in the pattern. The FTO crystallization was higher in the wSG substrate, while the PVWG substrate presented the lowest one.

**Figure 8:**
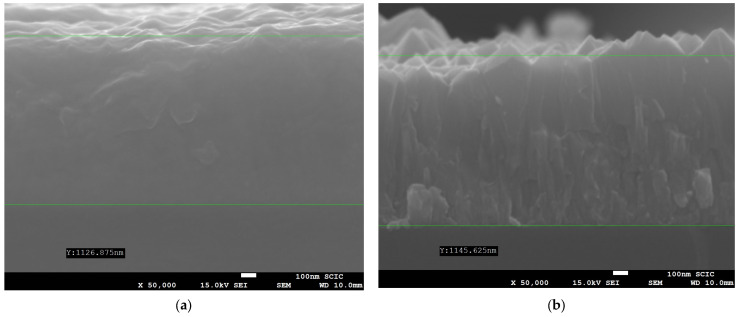
SEM images of deposited FTO: (**a**) PVWG; (**b**) wSG.

SEM micrographs of sputtered FTO on PVWG and wSG substrates are shown in Figure 8. The thicknesses of the films were 1126 and 1145 nm respectively, being sharper than the FTO grains in wSG, which is in concordance with XRD tests.

## 4. Discussion

Clean glass material was recovered from photovoltaic waste by a series of thermal processes and manual separation. PVWG was recovered from around 80% of the photovoltaic waste; this proportion is similar to the one reported by IRENA [2].

Ecological glass was formulated using the recovered glass by itself and combined with other waste materials, and glass substrates were prepared and characterized.

The compositions of PVWG and cSLG were comparable with that of typical SLG according to Hasanuzzaman et al. [36] with the composition range (70–75 wt% SiO_2_, 12–16 wt% of Na_2_O, and 10–15 wt% CaO), however, the Na_2_O content in PVWG was 0.6% higher, SiO_2_ was 1.13% higher, and CaO was 0.84% lower than in SLG. The increment of T_g_ and T_m_ of wSG in comparison to PVWG was due to the incorporation of SiO_2_ from quartz sand and CaO content from dolomite [37,38].

The transmittance in wSG was suitable compared to that of the commercial SLG, on the other hand, the transmittance of PVWG was higher than those of wSG and commercial soda lime glass. This phenomenon occurs due to the iron content in the cSLG and wSG glasses, which impacts in the absorbance of glass [39,40], and hence in the fraction of transmitted light across the substrate. According to Vogt et al. [40] in commercial soda-lime glass, the iron content is 0.093% while the iron content in “low iron soda-lime glass” destinated to photovoltaic application is around 0.02%. The produced glass substrates from different wastes exhibited a stable 80% transmittance similar to that of the commercial SLG.

The deposited FTO presented the main orientation in the plane (200) according to XRD results, this preferential orientation was observed by Muniramaiah et al. [41] for FTO deposited by spay pyrolysis. The deposited FTO material on the synthesized glass substrate from photovoltaic glass and other industrial wastes presented improved parameters such as sheet resistance and crystallization, compared from that deposited on commercial soda-lime glass. In the case of sheet resistance, the result of wSG was comparable with commercial FTO soda-lime glass substrate, with a laminar resistance of 9.39 ± 0.38 Ω/□ [42]. On the other hand, the deposited FTO on PVWG substrate presented a higher sheet resistance (10.13 ± 3.47 Ω/□) in comparison to that deposited on cSLG (8.48 ± 3.67 Ω/□), and the lowest crystallization grade in comparison to that exhibited on the other substrates, therefore the performance of the PVWG as a substrate was not satisfactory in this study.

## 5. Conclusions

Glass substrates using photovoltaic waste glass and other industrial wastes were synthesized and their possible usage in photovoltaic applications was evaluated. The glass substrate prepared only from photovoltaic glass waste presented the highest transmittance (90.15 ± 0.98%), however, it presented the highest sheet resistance (10.13 ± 3.47 Ω/□) and the lowest FTO crystallization in comparison to those of the other glass substrates, so its usage in thin film photovoltaic devices is not recommended in this study. On the other hand, the glass substrate prepared from photovoltaic glass waste and other residues presented a transmittance of 83.60 ± 1.52%, which is similar to that of commercial soda-lime glass (84.76 ± 3.60%), the lowest sheet resistance (7.84 ± 3.11 Ω/□) and the highest FTO crystallization in comparison to those of the other glass substrates including commercial SLG (8.48 ± 3.67 Ω/□); so their usage in the development of thin film photovoltaic devices could be considered. In general, an alternative process to incorporate photovoltaic waste glass and other industrial wastes in the production of glass substrates destinated for the development of thin film photovoltaic windows was proposed in this work.

Further investigation is focused on the approach of these waste materials in the development of glass substrates with improved properties for photovoltaic applications. In the case of PVWG glass substrate, although the poor performance of the FTO deposited onto it, its high transmittance is useful for thin film photovoltaic systems; therefore, the next research topic will be focused on the study of the chemical interaction between the glass substrate and the deposited TCO.

On the other hand, due to the good performance found in the glass substrate from photovoltaic glass and other wastes, such as acceptable transmittance and high potential for electron conductivity, further works will be carried out on the development of a thin film photovoltaic cell.

## Figures and Tables

**Table 1:** Wastes relationship used for wSG.

Wastes	Wt. (%)
PVWG	48.08
Quartz Sand	15.86
Dolomite	36.06

**Table 2:** Materials recovered from photovoltaic waste.

Material Recovered	Wt. (%)
PVWG	80.83
Poly-Si cell chunks	2.95
Tedlar^®^	11.95
Evaporated EVA	4.27

**Table 3:** XRF Analysis of PVWG and SLG (Wt. %).

Component	PVWG	SLG	wSG
SiO_2_	71.63	70.5	70.9
Na_2_O	14.20	13.6	7.18
CaO	8.98	9.82	10.68
MgO	3.58	3.9	4.91
Al_2_O_3_	1.13	1.4	5.65
SO_3_	0.17	N.D.	N.D.
K_2_O	N.D.	0.39	0.41
TiO_2_	N.D.	N.D.	0.27
L.O.I *	0.29	0.39	0.44
Total	99.98	100.00	100.44

* Loss on ignition.

## Data Availability

Not applicable.
